# RNA Virus Fidelity Mutants: A Useful Tool for Evolutionary Biology or a Complex Challenge?

**DOI:** 10.3390/v10110600

**Published:** 2018-11-01

**Authors:** Tiffany F. Kautz, Naomi L. Forrester

**Affiliations:** 1 Department of Microbiology and Immunology, University of Texas Medical Branch, Galveston, TX 77555, USA; kautz@bcm.edu; 2 Department of Pathology, University of Texas Medical Branch, Galveston, TX 77555, USA

**Keywords:** virus evolution, fidelity, vaccine, quasispecies

## Abstract

RNA viruses replicate with low fidelity due to the error-prone nature of the RNA-dependent RNA polymerase, which generates approximately one mutation per round of genome replication. Due to the large population sizes produced by RNA viruses during replication, this results in a cloud of closely related virus variants during host infection, of which small increases or decreases in replication fidelity have been shown to result in virus attenuation in vivo, but not typically in vitro. Since the discovery of the first RNA virus fidelity mutants during the mid-aughts, the field has exploded with the identification of over 50 virus fidelity mutants distributed amongst 7 RNA virus families. This review summarizes the current RNA virus fidelity mutant literature, with a focus upon the definition of a fidelity mutant as well as methods to confirm any mutational changes associated with the fidelity mutant. Due to the complexity of such a definition, in addition to reports of unstable virus fidelity phenotypes, the future translational utility of these mutants and applications for basic science are examined.

## 1. Introduction

It is estimated that there are greater than 320,000 viruses that infect mammals, the vast majority of which are unknown [1]. This in addition to recent metagenomic studies [2,3,4] suggests that only the smallest tip of the RNA virosphere has been identified [5]. With the exception of viroids [6], RNA viruses possess the highest mutation rate of all known species, ranging from 10^−4^–10^−6^ mutations per round of genome replication [7,8,9]. This is several orders of magnitude higher than DNA viruses [8] and thousands to tens-of-thousands of times higher than the mutation rates exhibited by bacteria and eukaryotes [10,11]. While the high mutation rate of RNA viruses generates many deleterious mutations, the abundance of RNA viruses suggests that the benefits of a high mutation rate may outweigh the risks. Therefore, it is important to understand what drives this evolutionary strategy and why it is so successful.

Most viral RNA-dependent RNA polymerases (RdRps) generate an estimated 1 mutation for every 10,000 bases, which is the approximate genome size of many RNA viruses [8]. The cloud of virus sequence diversity produced during RNA virus replication is commonly termed a quasispecies, defined as a collection of closely related virus variants, which are hypothesized to cooperate together to produce the overall virus phenotype [12,13]. One consequence of the high mutation rate associated with RNA viruses is that most mutations act as deleterious or neutral depending on the effects on RNA secondary structure or protein sequence. However, the large population sizes produced during the rapid population growth of RNA viruses are believed to result in an overall effective strategy for swift adaptation to changeable environments [14].

**Figure 1:**
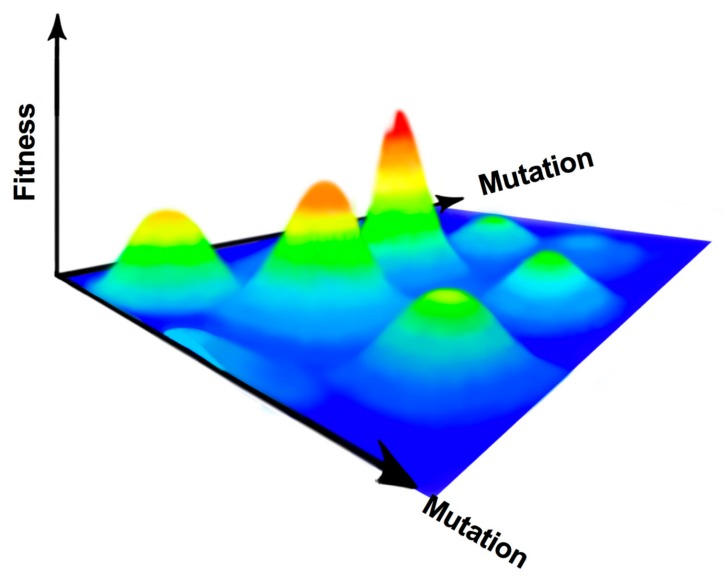
RNA virus mutational spectrum. As RNA viruses mutate, the sequence space is sampled to find areas of higher fitness. This space is hypothesized to be unstable, so it is best for mutation-prone RNA viruses to exist in flatter areas of this space where decreases in fitness are slight.

The mutational spectrum for an RNA virus can be visualized as a 3D field, with multiple peaks and valleys representative of virus fitness (Figure 1). The virus consensus sequence forms the foundation of the fitness landscape, allowing the virus population to explore this landscape through genetic drift with the prospect of finding and climbing new fitness peaks [15]. This landscape is not fixed but changes throughout the viral lifecycle, with the magnitude, location, and frequency of the peaks and valleys moving through both time and space [13,16]. Due to the high error rates produced during RNA virus replication, it is hypothesized that the best strategy for RNA virus survival is to produce a sequence foundation composed of low rolling hills, termed survival of the flattest [17,18]. Thus, mutations away from the consensus sequence should not have large, detrimental effects as the sequence space is explored, but still allow for the virus to identify fitness peaks to aid in survival during an unstable environment. However, survival is not guaranteed, and the mechanisms that allow for the ubiquity of RNA viruses can also be used against them.

RNA viruses are believed to exist on the edge of an error threshold, such that any increase in the mutational burden will cause the extinction of the virus population [15]. This error threshold is difficult to define, and likely dependent upon the environment, making the exact definition even more difficult, if not impossible [16]. Pushing virus populations over this error threshold has been examined empirically by exposing RNA viruses to various mutagens, most commonly with Ribavirin, a nucleoside analog that is able to pair with cytosine or uracil. As the concentration of a nucleoside analog is increased, so does the virus mutation frequency, until finally the population succumbs to the pressure and becomes extinct [19,20,21,22]. Recently, coxsackievirus B3 and influenza virus mutants were engineered to be 1 mutation away from a stop codon for each leucine or arginine codon. These mutants replicated identically compared to the unaltered viruses in vitro. However, when exposed to nucleoside analog treatment, these viruses were very sensitive, with sharp decreases in virus titer, demonstrating how close RNA viruses are to the edge of a bearable mutation burden [23].

Work with RNA virus fidelity mutants also suggests that error catastrophe can occur without pressure from a nucleoside analog. In the study that mutated coxsackievirus B3 to be more likely to produce stop codons, drops in titer were even more dramatic when a low-fidelity mutation was inserted into the genome, resulting in very little infectious virus [23]. Additionally, other studies have found that low-fidelity mutants subjected to bottleneck passaging can quickly become extinct, providing further evidence of an error threshold [24,25].

While work with fidelity variants of RNA viruses has been useful in defining the evolutionary precariousness of RNA viruses [23,24,25], there have been inconsistent phenotypes observed in the literature, suggesting that virus fidelity is more complex than previously believed. Here we review the latest literature and current hypotheses on the nascent field of RNA virus fidelity, as well as questions to pursue in the future.

## 2. RNA Virus Fidelity

### 2.1. Defining a Fidelity Mutant

The most basic definition of a RNA virus fidelity mutant is a virus that produces more or less error when replicating relative to the original virus. While identifying a fidelity mutant may appear straightforward, it can be difficult to link specific mutations in the virus genome to changes in fidelity. Additionally, questions arise that are difficult to answer. For example, is a virus a fidelity mutant if it is not high or low-fidelity for all mutation types? For half of the mutation types? As long as the overall balance is consistently higher or lower than the control virus? For example, a Ribavirin resistant foot-and-mouth disease virus variant was identified that lowered the overall virus mutation frequency, but only in the presence of Ribavirin [26]. While there was no difference in the overall mutation frequency in the absence of Ribavirin, the types of mutations produced were significantly different. Is this mutant a fidelity mutant or a more akin to a close cousin?

The answer likely depends upon the purpose of the project. For example, if the goal is to cause further attenuation of a vaccine by changing the fidelity, this is not likely to be important as long as the attenuation is stable. However, this information is liable to be important if a vaccine phenotype is not stable or when asking basic science questions. Unfortunately, the frequency of different mutations (e.g., transversion vs. transition mutations) is not available for most fidelity mutants, and the majority of those mutants for which this is available either find an imbalance in different types of mutation [27,28,29] or do not provide enough information to determine this [30,31]. Fortunately, this should be relatively easy to address in future fidelity work. Currently, most researchers have used the landmark poliovirus (PV) fidelity mutant studies as a benchmark to determine if they have discovered a virus fidelity mutant of their own.

### 2.2. Poliovirus

The first and most famous RNA virus fidelity variant was discovered by passaging PV in the presence of the nucleoside analog, Ribavirin [32]. During this passaging, multiple synonymous transition mutations appeared in the RdRp, as well as one nonsynonymous mutation, G64S. When the G64S mutation was inserted into the PV genome, this mutant was found to grow as well as the wild type (wt) virus in vitro. It was also resistant to multiple nucleoside analogs, and was much slower to revert a sequence necessary for PV guanidine hydrochloride resistance, all suggestive of a high-fidelity virus. Although the mutant grew well in vitro, G64S PV was slightly, but consistently outcompeted by the wt virus when passaged together [32]. Later, it was reported that this mutant was attenuated in vivo using poliovirus receptor (PVR) transgenic mice, as G64S PV was able to grow well in the muscle when injected intramuscularly (IM), but severely restricted in its ability to disseminate into the brain [33]. This was not caused by an inability to grow in these tissues, however, as mice infected intracranially (IC) were completely susceptible to infection by G64S or wt virus.

Soon after these reports, Vignuzzi et al. [34] independently recovered the G64S mutant, also by passaging PV in cells cultured with Ribavirin. The G64S mutant acted very similarly in the hands of a different lab, with no alteration of growth kinetics in vitro, as well as reduced lethality in vivo and restricted tissue tropism, with G64S virus unable to infect the central nervous system when introduced by the IM route. Confirmation of high-fidelity genome replication was performed by reverse transcriptase PCR (RT-PCR) of passage 3 virus stock followed by high throughput TOPO cloning and sequencing of a fragment of the virus genome. This resulted in the G64S virus population producing a 5.6-fold lower mutation frequency than the wt virus. Most intriguingly, artificial expansion of the G64S virus quasispecies caused this virus to increase in lethality while still maintaining the G64S mutation. However, when virus isolated from these mice was used to infect naive PVR mice, the G64S mutant was attenuated once again. This experiment provided very strong evidence for the quasispecies theory of RNA virus evolution by demonstrating that it was the diversity in the population that caused severe virulence in vivo, not any single mutation.

Later, Vignuzzi et al. [35] demonstrated that other amino acids in this position were also able to increase fidelity, such as G64A and G64T, albeit to a lesser extent than G64S. These initial studies with PV provided a strong prototype for future fidelity variants to be compared against.

### 2.3. Common Characteristics of Fidelity Mutants

In the years since the identification of high-fidelity PV, a large number of fidelity variants have been identified from a diverse group of RNA virus families, including Picornaviridae [24,25,27,28,33,34,36,37,38,39,40,41,42,43], Togaviridae [44,45,46,47], Flaviviridae [29,48], Caliciviridae [49], Arteriviridae [50], Orthomyxoviridae [31,51], and Coronaviridae [52,53]. The first fidelity mutants were identified similarly to the high-fidelity PV mutant by passaging virus multiple times in the presence of a nucleoside analog, usually Ribavirin. Over the course of passaging, the initial drop in virus titer caused by the nucleoside analog treatment would rise as the virus adapted to the conditions. When this occurred, the full virus genome or RdRp would be sequenced until promising mutations appeared. Sequencing usually revealed that many mutations had occurred, and these would be inserted individually or as a collection into the original virus using reverse genetic techniques and tested for fidelity perturbations. This is a rather laborious process with no guarantee of success, so most fidelity mutants have been identified by rationally targeting conserved residues that were previously implicated as fidelity regulators. Using these different techniques, over 50 fidelity mutants have been identified and characterized. Although the virus families are diverse, fidelity mutants share many common characteristics.

One necessary test for altered fidelity is to examine resistance to different nucleoside analogs. High-fidelity viruses are resistant to multiple nucleoside analogs, likely due to decreased nucleoside mis-incorporation, which correlates with higher fidelity overall. Low-fidelity mutants are less straightforward. Low-fidelity mutants generated by mutating residues important for high-fidelity are more sensitive to nucleoside analog treatment, regardless of the analog. However, low-fidelity mutants identified during nucleoside analog passaging are resistant to the nucleoside analog used for the identification, but are not necessarily resistant to other analogs [28,29,31,47].

Fidelity mutants generally do not exhibit attenuation during 1-step growth curves, which are typically performed using HeLa or BHK cells. The only clear exceptions to this are arbovirus low-fidelity mutants [29,46], which exhibit significant attenuation in mosquito cells but not vertebrate cells, and the PV fidelity mutants [27,33], which were attenuated in primary cells but not HeLa cells (although recent work has demonstrated a growth defect for PV G64S [54]). Further work is needed to determine if other fidelity mutants are also attenuated in primary cells. Additionally, as replication kinetics assays may not be sensitive to small differences between viruses (e.g. due to slight differences in timing the beginning of the virus infection, collection times, and pipetting errors, it is difficult to determine if 2–3-fold differences in titer are accurate between different viruses), a biochemical assay can be used to determine the exact rate of nucleoside triphosphate (NTP) addition (further detailed in “molecular and structural determinants underlying fidelity”). In brief, high-fidelity mutants incorporate NTPs at a slower rate, but more accurately [29,37,42,55], while low-fidelity mutants incorporate NTPs at a faster rate, but with more mistakes [24,25,29,42]. These differences are sometimes severe enough to change the specific infectivity of a virus (i.e., the ratio of virus RNA to plaque forming units (PFU)) [25,29,36,46,52,53] or relative fitness [27,31,32,34,41,43,44,53], but not always.

**Figure 2:**
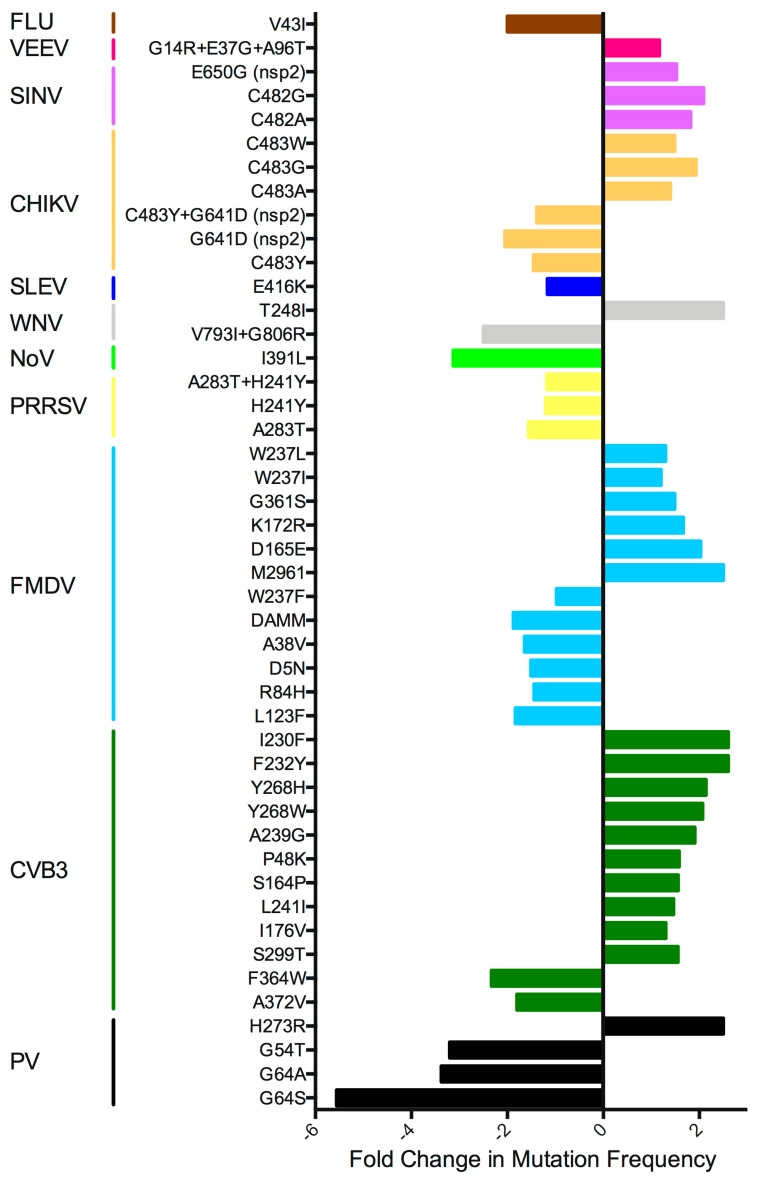
Fold-change in fidelity mutant mutation frequency relative to the wt virus. Most estimates are from TOPO cloning experiments, but if this information was not available, fold-change NGS data was used. Fidelity-altering mutations found outside the RdRp gene are indicated. Coronavirus data was excluded due to exceptional differences during RNA replication compared to the other RNA viruses. HEV71 data was excluded as mutation frequency was only reported for virus in the presence of ribavirin. FLU: influenza virus; VEEV: Venezuelan equine encephalitis virus; SINV: sindbis virus; CHIKV: chikungunya virus; SLEV: St. Louis encephalitis virus; WNV: West Nile virus; NoV: norovirus; PRRSV: porcine reproductive and respiratory syndrome virus; FMDV: foot-and-mouth disease virus; CVB3: coxsackievirus B3; PV: poliovirus.

To quantitatively calculate fidelity changes, virus RNA is usually isolated from virus stock or low passage virus, ranging from 1 to 5 passages. Most groups have measured mutation frequency as in the early PV studies, using RT-PCR followed by TOPO cloning and Sanger sequencing of the clones to screen for variants. Next generation sequencing (NGS) analysis has improved dramatically since these first studies, so newer publications also include data showing the amount of minority variant diversity generated across the entire virus genome. However, due to differences in NGS pipelines and diversity calculations, as well as differences in the number of virus passages, it is difficult to compare mutation frequencies from different papers. This is further complicated by the use of virus stocks grown in different cell types, as well as different passaging conditions, making it difficult to tease apart mutations promoted by selection versus inherent differences in the production of mutations. However, most fidelity mutants appear to produce ±1.5–2-fold change in diversity compared to wt virus (Figure 2). Although these differences in diversity appear small, these alterations have been shown in multiple viruses to translate to significant attenuation in vivo [24,25,27,33,35,37,39,41,42,43,44,46,47,50,51]. However, there is some debate as to the reasons for this observed attenuation [54].

Increased survival rates and tissue restrictions are commonly observed during the infection of hosts with fidelity mutants. It is currently not well understood why certain organs fail to become infected with fidelity variants, especially when different routes of inoculation are used, but this is likely due to increased virus sensitivity to host bottlenecks. Interferon (IFN) may also be a factor in this attenuation, but this was only observed for a high-fidelity/low-recombination PV mutant [56]. Alternatively, low fidelity mutants are likely attenuated in vivo due to an over abundance of deleterious mutants. Additionally, arbovirus fidelity variants are usually more attenuated in the mosquito vector than the vertebrate host, with lower virus titers or even almost complete restriction [29,44,46]. This pressure can be intense enough to select for complete reversion of the fidelity altering mutation, as observed for the low-fidelity chikungunya virus (CHIKV) and sindbis virus (SINV) mutants [46], suggesting that optimal virus diversity is especially important in the invertebrate vector.

### 2.4. Molecular and Structural Determinants Underlying Fidelity

To date, most fidelity mutants have been identified in the viral RdRp. The only exceptions are the coronavirus (CoV) ExoN mutants [52,53] and a mutation in the alphavirus helicase gene, nsP2, found to increase CHIKV fidelity [45]. Coronaviruses are members of the Coronaviridae family, some of which have evolved uniquely large RNA genomes (i.e., >20 kb). The ability of these large RNA viruses to persist is credited to the ExoN gene, which is a proofreading exoribonuclease [57]. When this proofreading function is ablated, RdRp fidelity drops by approximately 20-fold [52,58]. Interestingly, when a mouse hepatitis virus ExoN mutant was passaged hundreds of times, fidelity slowly increased, likely due to compensatory mutations that occurred in ExoN, as well as the RdRp, but reversion of the original ExoN mutations never occurred [53].

Of the fidelity mutants that induce changes in the RdRp, structural analyses show negligible effects on RdRp structure. Instead, changes in fidelity appear to be mediated by alterations in RdRp kinetics and molecular dynamics [59]. However, this is not known for all fidelity mutants, as some virus genera still lack a verified RdRp structure (e.g., the alphavirus genera).

**Figure 3:**
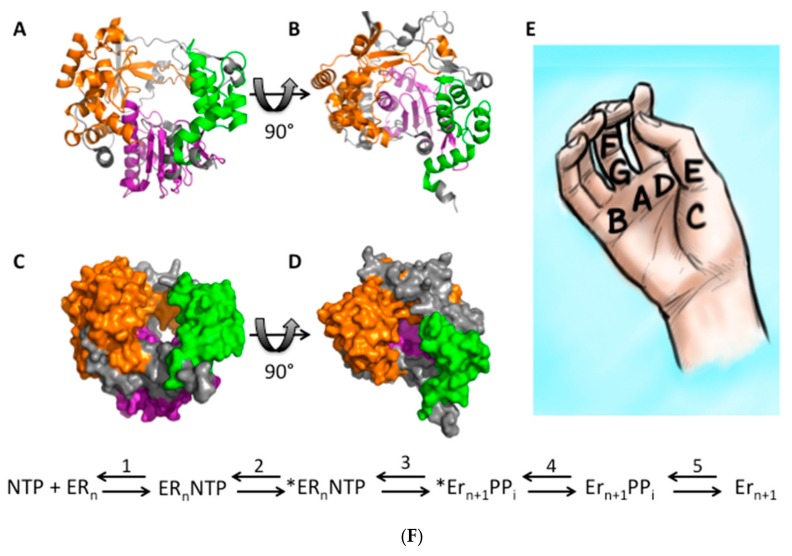
RdRp structure and RdRp kinetics. The FMDV RdRp structure [64] was downloaded from the RCSB PDB and visualized in PyMol v1.8.4.0 [65]. Structure (**A**,**B**) and surface models (**C**,**D**) are depicted. The RdRp is color-coded with the fingers as orange, the palm as purple, and the thumb as green. Location of conserved RdRp motifs as visualized on a right hand diagram (**E**). RdRp kinetics steps for NTP addition to an RNA chain (**F**). First, an NTP binds the RdRp, which causes a conformational change “*” (step 2) and activates the RdRp (step 3). The NTP is then added to the RNA chain, leading to another conformational change and translocation of the RNA (step 4). Finally, the PP_i_ leaving group is released (step 5), allowing the cycle to begin again.

RdRp NTP addition can be broken down into 5 steps (Figure 3F) [59]. First, an NTP binds the RdRp (step 1), which causes a conformational change (step 2) and activates the RdRp (step 3). The NTP is then added to the RNA chain, leading to another conformational change and translocation of the RNA (step 4). Finally, the pyrophosphate leaving group is released (step 5), allowing the cycle to begin again. Steps 2 and 4 are rate-limiting, so fidelity changes are believed to only alter these two steps.

Fidelity-altering mutations are usually found in motifs A and D of the RdRp active site (Figure 3A–E) [24,25,36,37,42], although other regions of the RdRp have been implicated in modulating fidelity. Mutations in the active site appear to alter fidelity by acting on the rate of polymerase incorporation, not the rate of NTP selection [28,60,61].

Unlike RdRp mutations found in the polymerase core, the high and low-fidelity G64S and H273R PV mutants are found on the periphery of the RdRp protein in the fingers domain. The current mechanistic theory for high-fidelity PV is that the G64S substitution interacts with other conserved residues in the RdRp active site, specifically motif A, to hydrogen bond to and orient the incoming NTP. The G64S mutation causes a misalignment of this hydrogen bond, which causes the misalignment to be even more severe when an incorrect NTP binds, thus increasing RdRp fidelity [62]. Alternatively, low-fidelity H273R PV decreases fidelity by favoring an open state of the RdRp, which decreases the duration of this fidelity checkpoint, thus increasing the likelihood of NTP misincorporation [63].

### 2.5. Fidelity Mutants as Live-Attenuated Vaccines

RNA virus fidelity mutants are believed to be promising vaccine candidates due to their increased in vivo attenuation and therefore an increased safety profile. High-fidelity vaccines for RNA viruses are of particular interest, because reducing the naturally high replication error rate should also increase the genetic stability of the vaccine. However, few of the above described fidelity mutants have ever been tested as vaccines.

Early in the fidelity variant literature, high-fidelity G64S PV was tested for its genetic stability and efficacy as a vaccine [35,66]. To examine stability, the microRNA (miRNA) let-7 was inserted into the PV genome, and cells expressing the miRNA were infected with wt or high-fidelity PV. The wt PV quickly mutated this miRNA sequence and began a productive infection, while the G64S variant necessitated an additional 48 h before it was also able to overcome this selection pressure. Interestingly, all of the G64S isolates contained the same deletion to remove the let-7 miRNA, while the wt virus isolates reverted using different mutations and deletions. This suggests that although high-fidelity viruses are able to revert that these reversions are predictable and can thus be anticipated and prepared for. When tested as a vaccine, the G64S mutant was shed approximately 100-fold less in feces than the wt virus. Additionally, G64S vaccinated mice produced slightly, but not significantly higher neutralizing antibody titers, and were fully protected against challenge with wt PV.

A low-fidelity PV mutant, H273R [27], was also found to provide complete protection when used as a vaccine, but only after a high dose of 10^7^ PFU, a dose which resulted in the paralysis of approximately 10% of vaccinated mice. Neutralizing antibody titers were correlated with protection, but the fidelity mutant appeared to require a 100 to 1000-fold higher dose to produce a similar neutralizing antibody titer to the wt virus. While it is unknown whether H273R is prone to reversion, this mutant was shown to be sensitive to bottlenecks, undergoing population extinction at a 10-fold higher PFU than the wt virus. This suggests that additional attenuating mutations would be necessary to improve the safety of this vaccine, but only if these mutations would not further reduce immunogenicity, necessitating a higher vaccine dose.

More recently, a low-fidelity version of a live-attenuated vaccine candidate for Venezuelan equine encephalitis virus (VEEV), TC-83, was identified [47]. The low-fidelity version of this vaccine was significantly more immunogenic than the wt vaccine and was able to protect against challenge with wild type virus. This mutant also displayed attenuation, albeit inconsistently, in an infant mouse model. Unlike the wt TC-83 vaccine, which typically reverts back to wt VEEV virulence after 2–3 infant mouse passages [67], the low-fidelity mutant was unable to revert to wildtype virulence, even after 10 in vivo passages. Importantly, despite its low-fidelity genotype, there was no reversion of the fidelity-altering mutations during this passaging, and, despite its increased mutation frequency, a decreased incidence of reversion of the mutations responsible for the attenuation of TC-83. This low-fidelity vaccine candidate demonstrated that combining fidelity-altering mutations with other attenuating mutations could be a viable live-attenuated vaccine strategy.

Lastly, a low-fidelity mouse-adapted severe acute respiratory syndrome (SARS) CoV ExoN mutant was examined as a potential vaccine candidate [66]. Removal of the ExoN gene increased the virus mutation frequency by 11.5-fold without harming replication kinetics in vitro [66]. This decrease in fidelity was stable, with a much higher accumulation of virus mutations during infection of mice with the ExoN mutant versus the wt virus. Even after 3 murine passages, the ExoN mutant virus remained attenuated, while the wt virus became completely lethal after 1 passage. As a vaccine, the mutant was well tolerated, even in immunocompromised hosts. The ExoN mutant was also able to protect against challenge and stimulated a neutralizing antibody response. However, how this compares to the wt virus or any other vaccine is unknown.

### 2.6. Are Some Fidelity Mutants Actually Kinetic Mutants?

Theoretically, fidelity mutants should not be attenuated in vitro, because changes in fidelity should not be detrimental to virus replication [68], at least according to the initial fidelity mutant studies. The lack of a growth defect is a desirable characteristic for translational research, such as the generation of live-attenuated vaccines. However, many fidelity mutants do grow slightly slower than wt virus [38,41,46,49,69], especially in primary vertebrate cells [33,42] and mosquito cells [29,46,48]. In fact, recent research has clearly demonstrated that the high fidelity G64S PV mutant has a significant growth defect [54].

While the mechanisms behind this slight attenuation are not well understood, this suggests that in a more complex environment, such as those found in vivo, that fidelity mutants may essentially behave as kinetic mutants [33]. As fidelity mutants commonly display lower titers and tissue restriction in vivo [25,34,35,39,44,46,50,70], perhaps the lower titers produced by fidelity mutants act as a bottleneck, producing a virus population without the correct mutant spectrum to infect the same number of tissues as wt virus. Additionally, a drop in the virus titer due to slightly slower replication kinetics likely makes the infection easier for the host to control, which could be a confounding factor in the quasispecies hypothesis of attenuation commonly believed to be responsible for the in vivo attenuation observed for fidelity mutants. This begs the question of whether replication fidelity and kinetics can ever truly be separated.

Newer technologies may aid in determining the differences between fidelity and replication if they do indeed exist. For example, measuring the incorporation of 1–2 nt, as is common in sym/sub replication kinetics experiments, is not an accurate depiction of virus replication, which naturally requires the RdRp to replicate not one, but thousands of nucleotide bases. A new technique, known as magnetic tweezers, addresses this by attaching a coverslip to one strand of an approximately 3 kb double-stranded RNA molecule and a magnetic bead to the other [71,72]. As the RdRp replicates the RNA, this releases the beaded RNA strand from the anchored RNA strand, allowing the magnetic bead to migrate upwards towards a magnet. The longer the distance between the coverslip and the magnet, the more efficient the RdRp is at replication.

Sym/sub replication studies have shown low-fidelity viruses to be fast, but inaccurate NTP incorporators [24,25,29,42]. However, magnetic tweezers have shown that low-fidelity PV H273R also displays an increased number of pause events, which are associated with the incorporation of incorrect NTPs [71]. This suggests that low-fidelity polymerases are prone to stuttering when replicating, which may impact other polymerase-associated events, such as recombination. Understanding the role of RdRp processivity and its ties to fidelity is one step towards closing the gap of knowledge between what is observed during very simple measures of virus replication, such as sym/sub replication, and what is observed in cell culture and animals.

For some viruses, such as those in the alphavirus genus, the RdRp cannot be isolated due to instability, and thus fine measurements of RdRp replication kinetics are not possible. Single-cell sequencing presents an exciting alternative as it also offers a better understanding of the fate of each virus-infected cell [73,74,75]. Interestingly, when low-fidelity H273R PV was examined using this technique, it was found to cause a reduced number of infections compared to wt virus, as well as a delay in replication initiation [73]. However, H273R was able to replicate quickly enough to reach peak titer at the same time at wt PV. This experiment confirmed what had been measured using the sym/sub system [27], suggesting that some of the measurements that once required cell-free systems to assay can now be performed in cells. This also improves upon the more standard biochemical and growth curve kinetics assays by allowing for the measurement of thousands of infections at the same time in replicate, instead of only examining a composite. However, as these techniques are relatively new, this type of analysis is not yet widely available.

### 2.7. Fidelity Mutant (In)stability

While fidelity mutants exhibit certain trends, like attenuation in one environment versus another, these do not appear to always be stable. This is not entirely surprising, as RNA virus fitness landscapes are hypothesized to be turbulent [13,16], and there is a paucity of methods to aid in the prediction of these landscapes for most viruses. When examining the literature, most fidelity mutants have only been characterized in the paper showcasing their discovery, and those that have appeared in more than one publication are used to examine something novel. Occasionally, however, assays are repeated, and these commonly show inconsistent fidelity and/or phenotypes, as described below.

The high-fidelity human enterovirus 71 (HEV71) mutants were first described by Sadeghipour et al. in a pair of concurrent papers [38,70]. While G64R, G64T, and S264L RdRp mutations were all able to increase HEV71 fidelity in vitro [38], only S264L caused attenuation in vivo when using 5-day-old BALB/c mice, which have an intact IFN response [70]. However, when these mutants were later examined in a slightly different virus backbone that was not murine-adapted, G64R was approximately 3-fold less virulent than S264L when using 10-day-old AG129 mice (IFN-receptor deficient), which itself was 37-fold less virulent than the control virus [39]. This is odd, because these results suggest that small changes to the HEV71 backbone can cause dramatic changes in virulence. However, if the highly conserved PV G64 residue is able to increase fidelity in a distantly related enterovirus such as HEV71, this is not likely to be the cause of differences in virulence. Alternatively, changing the mouse model may have resulted in decreased G64R virulence in AG129 mice. However, as PV G64S [33,34] and other fidelity mutants [25,27,37,40,41,42,43,44,46,50,51] are attenuated in mice with intact IFN, it would therefore be predicted that the HEV71 G64R mutant would also be attenuated in the BALB/c model. Thus, there is no clear explanation for why the HEV71 G64R mutant would behave so differently in the hands of different labs.

Inconsistent attenuation of fidelity mutants in vivo is not limited to HEV71. A recently identified low-fidelity mutant for VEEV was also found to exhibit inconsistent attenuation [47]. While the initial low-fidelity virus stock was significantly attenuated compared to the wt TC-83 virus in an infant mouse model, a repeat of this experiment with two other independently-derived virus stocks failed to replicate this attenuation. This result suggests that innate differences in virus stocks could result in fluid phenotypes, but further research is needed to fully understand this phenomenon.

The most famous and highly studied fidelity mutant, high-fidelity PV G64S, is also not immune to incongruent results. The first study to examine PV G64S in vivo infected cohorts of 10-week-old PVR mice IM with 5 × 10^6^ PFU of G64S or wt PV [33]. Of the 25 mice per cohort, approximately 10 G64S PV mice exhibited no symptoms over the course of infection, while only 5 of the mice infected with wt PV exhibited no symptoms. While it was stated that fewer deaths were recorded for G64S infected mice, this data was not shown. Similar results to this initial paper were recently published by Fitzsimmons et al. [54], which found that only 10% of 6–8-week-old PVR mice infected IM with 10^6^ PFU G64S PV were able to survive infection, although time to death was delayed for these mice. Fitzsimmons et al. additionally reported IM median lethal dose (LD_50_) values of 1.47 × 10^5^ PFU for wt PV and 6.81 × 10^5^ PFU for G64S, a 4.6-fold difference. In contrast to these papers, Vignuzzi et al. [34] found that 100% of 8-week-old PVR mice infected IM with 10^7^ PFU of G64S were able to survive infection, suggesting that this G64S virus stock was more attenuated than the stocks used during these other in vivo virulence studies [33]. Vignuzzi et al. also used this experiment to calculate LD_50_ values, which were 1.2 × 10^6^ PFU for wt PV and 3.9 × 10^8^ PFU for G64S, a 325-fold difference, which were the same LD_50_ values published in a later paper also by the Vignuzzi lab [35].

This alone would not be too concerning as all of the above papers demonstrated that G64S PV was more attenuated in vivo than wt PV. However, a recent dissertation [76] reported complete hind limb paralysis in all mice (a common proxy for lethal infection) when 4–6-week-old PVR mice were infected IM with 10^7^ PFU of G64S. These mice also showed no difference in the LD_50_ values for the G64S mutant versus the wt PV. Intriguingly, for this group, G64S was only more attenuated than wt PV when 5-week-old mice were infected IP. This finding was further reinforced by Xiao et al., which also found that 5-week-old PVR mice infected IM with 10^7^ PFU of the G64S mutant succumbed to infection at identical rates as the wt virus [30]. Additionally, there was no difference in the LD_50_ values. Xiao et al. suggested that this difference may be due to age, as the wt PV LD_50_ for 8-week old mice in this study was 10^7^ PFU versus 5 × 10^5^ PFU for 5-week-old mice. However, it is unknown how this translates to high-fidelity G64S PV, as this was not shown, and does not explain why the LD_50_ values would be so similar for wt PV and G64S. Again, as seen for HEV71 and VEEV, fairly large differences in phenotypes were observed when in vivo experiments were repeated.

The inconsistency observed for in vivo attenuation with HEV71, VEEV, and PV fidelity mutants suggests that this is a prevalent occurrence for fidelity mutants. This is concerning, as one of the best pieces of evidence for the theory of virus quasispecies is the original high-fidelity PV study by Vignuzzi et al., which demonstrated that it was the overall diversity of the quasispecies that caused virulence, not any one mutation [34]. Perhaps it is not surprising that in vivo attenuation is inconsistent, as it is hypothesized that high fidelity viruses are less likely, not entirely unable, to produce the mutants necessary for different tasks (e.g., IFN evasion, host switching). This is supported by recent studies that have correlated specific minority variant mutations to increases in virus fitness [77,78]. Additionally, while low-fidelity viruses are hypothesized to overproduce deleterious, and potentially interfering, mutants [79], perhaps some stocks harbor more interfering mutants than others, while others may generate more high fitness mutations due to random chance, leading to differences in attenuation. Future research examining this is greatly needed.

While the mechanism(s) behind this variation in attenuation in vivo have yet to be determined, a study by Xiao et al. hints that PV is able to overcome increased fidelity by using recombination, suggesting that recombination may be playing a compensating role for other fidelity mutants [56]. Additionally, as suggested by Xiao et al., the age of the mouse could explain the severe changes in attenuation, although this may be specific to PV G64S. If so, this is of large concern for vaccine development, as this suggests that, at best, only certain human populations would benefit from the increased vaccine attenuation granted by fidelity mutant vaccines, or at worst, that there would be no discernable difference between vaccines with or without altered fidelity. This needs to be examined closely to determine exactly what is causing these changes in virulence and if these mechanisms are conserved between different fidelity mutants. At a minimum, this suggests that separate LD_50_s should be performed for each altered fidelity mutant stock to ensure consistent attenuation.

**Figure 4:**
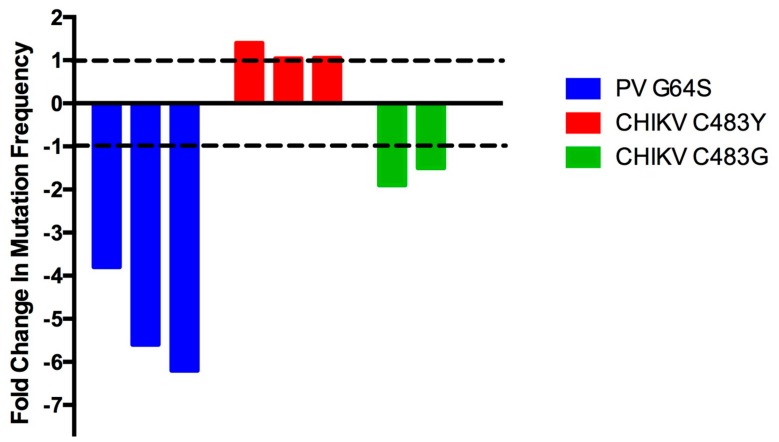
Variation in fidelity mutant mutation frequencies. Variation in the mutation frequency of unaltered virus versus: high-fidelity PV G64S [27,34,35], high-fidelity CHIKV C483Y [44,45,46], or low-fidelity CHIKV C483G [45,46]. Dashed lines indicate equal levels of mutation frequency for control and fidelity mutant viruses.

Additionally, the quantitative diversity of fidelity mutants can be somewhat inconsistent. For example, the mutation frequency of high-fidelity CHIKV C483Y was initially determined to be approximately 1.4-fold higher than wt virus [44]. Five independent virus stocks for each virus were used to determine these mutation frequencies, and, interestingly, there was large overlap of the mutation frequency error bars between the wt and high-fidelity mutant viruses. Later publications that used C483Y as a control found no difference in the mutation frequencies generated by C483Y or wt CHIKV [45,46]. Importantly, the mutation frequency per 10^4^ bases was slightly higher for wt CHIKV in the initial study compared to more recent papers (5.1 versus 4.4 and 4.4), as well as slightly lower for C483Y (3.6 versus 4.1 and 4.2), suggesting that slight fluctuations in both the wt and RdRp mutant stock mutation frequencies can reduce or entirely ablate significant differences in replication fidelity. Alternatively, this change in the CHIKV C483Y mutation frequency is perhaps explained by an nsP2 mutation, G641D, which was found during the initial high-fidelity CHIKV identification [44] and later shown to also increase CHIKV fidelity [45]. It would be interesting to determine the occurrence of this nsP2 mutation in different CHIKV C483Y stocks, and whether the appearance of this mutation in the minority variant population is correlated with decreased virus sequence diversity. Importantly, while the flux in this CHIKV mutant’s replication fidelity is likely one of the more egregious examples, some degree of mutation frequency flexibility is probably to be expected due to differences in mutation frequency measurements, random chance, and other unknown variables (Figure 4).

Unfortunately, for many fidelity mutants we may never know which aspects are or are not repeatable, as the majority of fidelity mutants have not been closely and repeatedly examined. This is problematic, as outcomes from experiments using fidelity mutants are being used to draw significant conclusions regarding RNA virus evolution [23,80]. Without a thorough understanding of the shortcomings of each proposed fidelity mutant, use of these fidelity mutants is likely to lead to ambiguous and misrepresentative results, especially if other controls are not used. This is especially concerning for the use of fidelity mutants as vaccine candidates, as these inconsistent in vivo outcomes represent a significant barrier against the development of fidelity mutants as live-attenuated vaccines. These inconsistencies necessitate further consideration when determining the defining characteristics of a virus fidelity mutant.

## 3. Conclusions and Future Directions

When the first fidelity mutants were identified in the mid-aughts, they were hailed as major breakthroughs in our understanding of virus evolution and as tools for understanding the role of viral diversity in virus transmission and pathogenesis. Since these initial reports there have been an explosion of fidelity mutants, which have been used in numerous studies to demonstrate that RNA viruses exist in a narrow range of mutational fidelity, and that any perturbation of this diversity results in attenuation in vivo. However, it is becoming clearer that the phenotypes of these mutants are not always stable, and that there are some caveats to the production of fidelity variants that need to be explored more widely.

RNA viruses containing putative fidelity mutants are generated from infectious clones. If the outcome of virus recovery is variable and leads to major shifts in the observed phenotype for a fidelity mutant, despite the same consensus level mutations being present, this creates a significant barrier for developing either a controlled experiment, or, in the case of vaccines, a controlled manufacturing process. Although using fidelity mutants translationally as vaccines held such promise during the initial high-fidelity PV studies, more recent evidence suggests that this method of virus attenuation is only workable if attenuated viruses are used as the backbones for these mutants, such as in the VEEV TC-83 study [47]. There is some historical evidence for this, as the vaccine for yellow fever, 17D, is believed to be a safe and effective vaccine based upon multiple attenuating mutations as well as a high fidelity polymerase [81,82]. For vaccines, at a minimum, correlates of attenuation need to be developed by generating many pools of virus stock, establishing a minimum threshold of attenuation (e.g., a significantly lower LD_50_ in a mouse model), and sequencing these stocks to determine sequences associated with attenuation, virulence, and immunogenicity. It would also be important to passage these stocks to guarantee stability of the vaccine genotype and phenotype to help reduce adverse events following vaccination. Other experiments with altered fidelity mutations should also include replicates from different viral stocks. Without this, it will be impossible to determine the consistency of the fidelity variant.

Perhaps in the future the instability exhibited by fidelity mutants will be overcome, allowing for these fidelity mutants to be used as more effective tools for basic science as well as live-attenuated vaccines. Until then, fidelity mutants will remain only a tool for basic science, at least when all the above caveats regarding their use are considered.
